# Concurrent Undernutrition and Overnutrition within Indian Families between 2006 and 2021

**DOI:** 10.1016/j.cdnut.2023.101987

**Published:** 2023-08-19

**Authors:** Laxmi Kant Dwivedi, Parul Puri, Anjali Pant, Alka Chauhan, Samuel Scott, Shrikant Singh, Sarang Pedgaonker, Phuong H. Nguyen

**Affiliations:** 1 International Institute of Population Sciences, Mumbai, India; 2 International Food Policy Research Institute, South Asia Office, New Delhi, India

**Keywords:** anthropometry, double burden of malnutrition, India, overweight, residence, underweight, wealth

## Abstract

**Background:**

The double burden of malnutrition (DBM), characterized by concurrent undernutrition and overnutrition, is a growing global concern. Families share resources and eating behaviors and programs often target households, yet evidence of the DBM at the family level is scarce.

**Objectives:**

This study examined trends and inequality in the intrahousehold DBM in India between 2006 and 2021.

**Methods:**

Data were from 3 waves of India’s National Family Health Survey (NFHS 2006, 2016, and 2021). We examined 3 types of household member (with children aged <5 y) combinations: mother–child (*N* = 328,039 across 3 waves), father–child, and parent (mother and father)–child (*N* = 47,139 for each pair). The DBM was defined as one or more individuals with undernutrition (either wasting or stunting in children or underweight in adults) and one or more overweight individuals within the same household. DBM was examined over time, at national and subnational levels, and by residence and wealth.

**Results:**

Nearly all DBM was in the form of an overweight parent and an undernourished weight or stunted child. The prevalence of parent–child DBM increased from 15% in 2006 to 26% in 2021. Father–child pairs experienced the most rapid DBM increase, from 12% in 2006 to 22% in 2021, an 83% increase, driven by increasing overweight among men. In 2021, the DBM was highest in North-Eastern and Southern states, and among relatively rich households from urban areas. The increase in the DBM was faster in rural areas and among poor households compared with that in urban areas and rich households. Urban–rural and rich–poor inequalities in the DBM have decreased over time.

**Conclusions:**

The intrahousehold DBM has increased over time, affecting 1 in 4 households in India in 2021. Family-based interventions that can simultaneously address child underweight and parent overweight are required to address India’s increasing intrahousehold DBM.

## Introduction

The United Nation Decade of Action on Nutrition seeks to eliminate malnutrition in all of its forms, including undernutrition, overnutrition, and their coexistence, known as the double burden of malnutrition (DBM) [1]. The DBM mainly affects low- and middle-income countries (LMICs) experiencing a nutrition transition [2] where food poverty is being replaced by high availability of refined and processed foods and physical activity is decreasing [3]. India is home to one-third (40.6 million) of the total number of stunted under-five children globally and 5% of Indian children aged 5–19 y are overweight [4]. It is projected that, by 2030, 1 in 10 obese children globally will be from India [5].

The DBM is often described at the individual (concurrent undernutrition and overnutrition in the same person) or population level (concurrent undernutrition and overnutrition between individuals belonging to the same population) [6], but very few studies have examined DBM within families, i.e. overnutrition in one family member and undernutrition in another family member from the same household. Two common scenarios of intrahousehold DBM in LMICs have been described [7]. The first is an overweight mother who has relatively high education and lives in an urban household with relatively high income, but her child is underweight; in this scenario, it is possible that the mother buys large quantities of readily available processed foods, is not physically active, and does not have time to breastfeed her child. The second common scenario is an overweight mother who has relatively low education and is from a poor household, whose child is underweight. This scenario exists in both rural and urban contexts and likely has complex mixed causes such as inability to purchase and/or being unable to access nutritious foods, poor nutrition knowledge leading to poor child feeding practices, or conservation of fatty tissue in mothers who were previously undernourished. Given that these scenarios exist in families, who share resources and behaviors, there is a need to address both undernutrition and overnutrition simultaneously and the World Health Organization has outlined a set of “double-duty actions” to this end [8].

Despite progress in various sectors in India, the persistence of undernutrition alongside rising overweight/obesity is still a significant health challenge and disproportionately affects certain segments of the population [9,10]. A substantial portion of the population still faces poverty and food insecurity [11], a condition related to inadequate access to sufficient, safe, and nutritious food, and has been linked to both undernutrition in children and overweight in women [[12], [13], [14]]. To improve targeting of double-duty actions in a diverse country such as India, a better understanding is needed regarding what types of the DBM exist between household members within families and how the prevalence of different DBM types varies by geographic location or socioeconomic strata. While many studies from LMICs have reported the DBM between mother–child pairs, very few have examined the issue among father–child or parent–child pairs, despite recognition that fathers play a crucial role in addressing the coexistence of under- and over nutrition [15,16]. Beyond being the primary earners, fathers actively contribute to their family members’ physical, emotional, and psychological well-being. Fathers can promote healthy eating habits and ensure access to nutritious foods, and thus can be a useful instrument in addressing both under- and over nutrition [[17], [18], [19]]. Therefore, the aims of the present study were *1*) to explore trends in the DBM over time for different types of household member pairs at national and subnational levels in India and *2*) to assess whether the change in the DBM between 2006 and 2021 in India varied by residence and wealth groups.

## Methods

### Data and study sample

The study employed nationally representative data from 3 National Family Health Surveys (NFHS-3 2005-06 [20], NFHS-4 2015-16 [21], and NFHS-5 2019-21 [22]) to capture trends over the past 15 y. The NFHS surveys are conducted under the stewardship of the Ministry of Health and Family Welfare, Government of India, and provide data on India’s population along with health and nutrition indicators. NFHS surveys are representative at the national and subnational levels.

**FIGURE 1:**
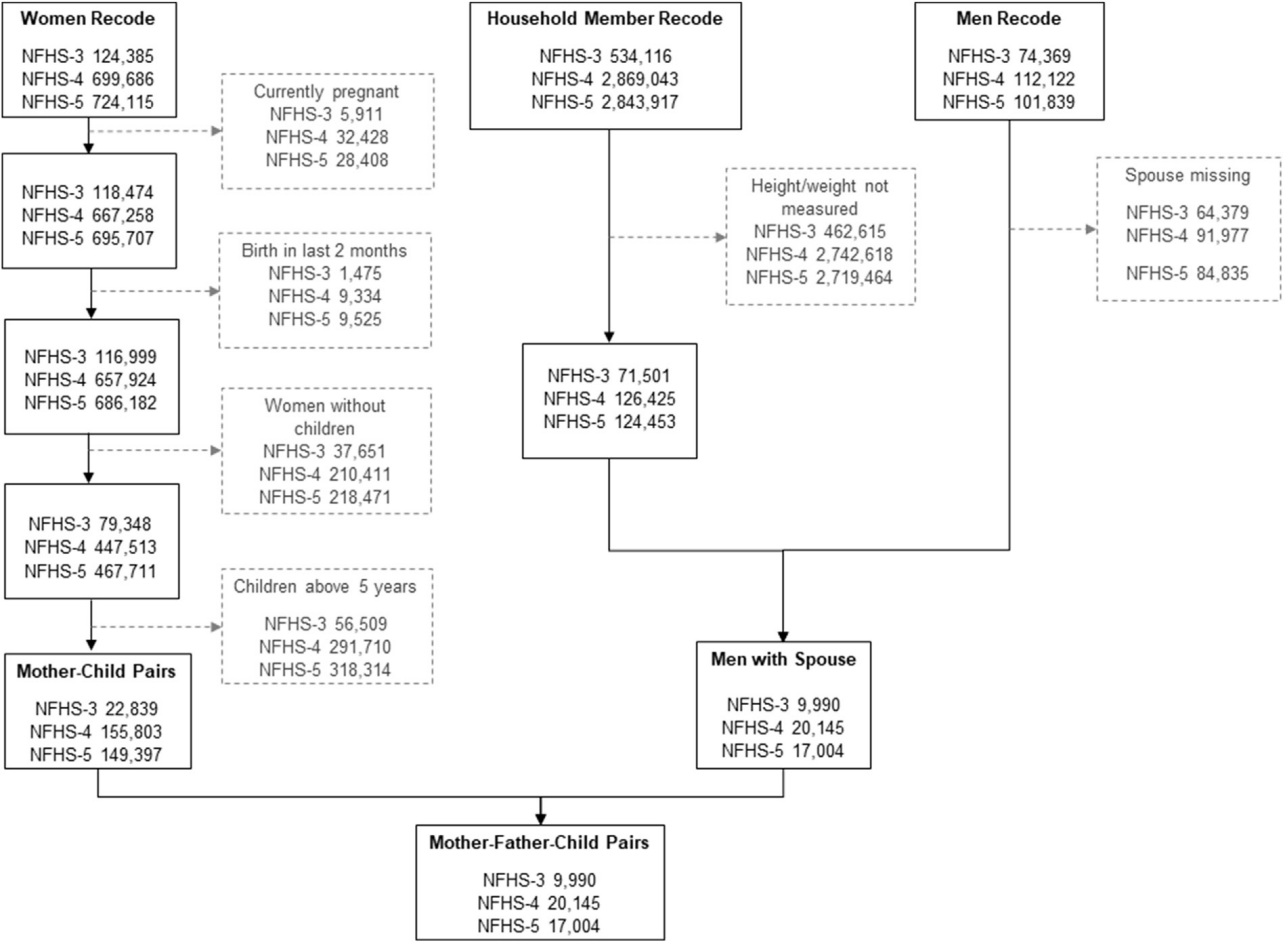
Flowchart depicting exclusion criteria, merging, and sample size for National Family Health Survey (NFHS), 2006-21. Boxes with gray text and dotted gray borders show sample exclusion steps.

In this study, we used data from households, men, women, and children from each NFHS round. For women, we restricted the sample to women aged 15–49 y who were not pregnant, did not give birth in the 2 mo before the survey, had at least 1 child aged <5 y (Figure 1). For men, we exclude those without data on weight and height. The final analytical sample of this study included 22,839 mother–child pairs and 9990 mother–father–child pairs in 2006; 155,803 mother–child pairs and 20,145 mother–father–child pairs in 2016; and 149,397 mother–child pairs and 17,004 mother–father–child pairs in 2021 (Figure 1).

### Anthropometric measurement

Anthropometric measurements were obtained by trained field staff using standard procedures [23]. Weight was measured using the Seca 874 digital scale. Height was measured using the Seca 213 stadiometer for adults and children aged 24–59 mo. Recumbent length was measured using Seca 417 infantometer for children <aged 24 mo [[20], [21], [22]].

For children, height and weight were then converted to *z*-scores (height-for-age [HAZ] and weight-for-height [WHZ]) using child age in century-day codes format across NFHS rounds. Stunting and wasting were defined as a HAZ or WHZ of <−2 SD from the median of the reference population, respectively. Overweight/obesity was defined as a WHZ of >2 SD [24]. For adults, height and weight were used to calculate BMI, and they were categorized as underweight (BMI < 18.5 kg/m^2^) or overweight (BMI > 23 kg/m^2^) based on recommendation for Asian population [25].

### Defining double burden of malnutrition in the context of India

The framework proposed by Davis et al. in 2020 was used to define the DBM [26]. The framework proposes 3 steps to build context-specific definitions of the DBM: (a) identifying the assessment level (population, household, dyad, or individual); (b) selection of target population; and (c) selection of malnutrition form. For undernutrition, anthropometric measures or measures of anemia and micronutrient deficiency can be considered. In the case of over nutrition, anthropometric measures or cardiometabolic risk measures can be considered. We chose to focus on anthropometric indicators for the current analysis.

We examined the DBM at the household level using 3 types of household member pairs: (a) father–child, (b) mother–child, and (c) parent–child (where parent could be either father or mother). The DBM was defined as one or more individuals with undernutrition (either wasting or stunting in any of the under-five children or underweight in adults) and one or more individuals with overweight or obesity within the same household [27]. All children resided in the same household as their parent(s). For example, if, in a household, the parent (mother or father) was overweight and any of their under-five children was undernourished (either wasting or stunting), we would consider the DBM to exist in this household. The DBM would also be present in a household with an underweight parent and at least 1 overweight child aged <5 y.

### Analytical approach

Descriptive analysis was used to examine temporal changes from 2006 to 2016 and from 2016 to 2021 for each type of household member pair. Weighted percentages of the DBM were computed to account for the cluster sampling design. Changes over time are presented as percent change and as average annual change rate (AACR) using *t*-statistics and *P* values to depict statistical significance.

Subnational variations in the DBM over time were visualized using bar graphs for selected household member pairs. Multivariable logistic regression models were applied to assess how the changes in the DBM differ by place of residence (urban and rural) and wealth quintile. The predicted prevalence for the 3 types of household member pairs was estimated using multivariable logistic regression after pooling the 3 rounds of NFHS data. The model was adjusted for covariates at child (age and sex of the youngest child), mother (age at birth of index child and education), and household levels (number of living children) along with survey period and its interaction with the selected covariates. Wealth quintile is a composite metric that gauges the overall standard of living of a household using a principal component analysis. It is computed using information on house and land ownership, housing structure (floor, wall, and roof materials), access to services (electricity, gas, water, and sanitation services), and ownership of several assets (car, motorbike, bicycle, television, radio, computer, refrigerator, watch, mobile phone, electric fan, bed, mattress, table, chair, press cooker, sewing, air conditioner, washing machine, animal-drawn cart, water pump, thresher, and tractor). Details of the methods used to measure wealth quintile are provided elsewhere [28].

Statistical analysis and data visualization were performed with STATA 17.0 (StataCorpTM) and RStudio 1.1.463 (R Studio, Inc.). All estimates were reported after accounting for complex survey design of the NFHS data and survey weight (women’s weight for women–child pair and men’s weight for father–child and parent–child pairs. The study followed the Strengthening the Reporting of Observational Studies in Epidemiology (STROBE) reporting guidelines for cross-sectional studies.

## Results

### Trends in double burden of malnutrition over time

The prevalence of undernutrition and overnutrition among children was presented in Supplemental Figure 1. The DBM among parents–children living in the same household was 15% in 2006, rising to 24% in 2016 and 26% in 2021 (Table 1). The AACR in the prevalence of the DBM was higher in 2006–2016 compared to 2016–2021 (5% vs. 2%, respectively). The prevalence of the DBM was higher in father–child compared to mother–child dyads (19% vs. 13% in 2016 and 23% vs. 15% in 2021). The rate of change for any DBM of parent–child pairs over time for was higher in 2006–2016 (AACR 5.4%) compared to 2016–2021 (AACR 2.3%).

**TABLE 1:** Distribution of various combinations of Double Burden of Malnutrition, National Family Health Survey, 2006–2021

Type of intrahousehold double burden	Prevalence, %			Change, %^1^		AACR, %^2^	
	NFHS-3 (2006)	NFHS-4 (2016)	NFHS-5 (2021)	2006–2016	2016–2021	2006–2016	2016–2021
Father–child pairs, *n*	9990	20,145	17,004				
Father underweights, child overweight	0.5	0.3	0.5	−49.0∗∗∗	73.1∗∗∗	−4.9	14.6
Father overweight, child undernourished^3^	11.2	18.6	21.4	66.0∗∗∗	14.9∗∗∗	6.6	3.0
Father any DBM, child any DBM	11.7	18.9	22.8	61.3∗∗∗	15.7∗∗∗	6.1	3.2
Mother–child pairs, *n*	22,839	155,803	149,397				
Mother underweight, child overweight	0.9	0.5	0.6	−43.7∗∗∗	22.5∗∗∗	−4.4	4.5
Mother overweight, child undernourished	7.5	12.4	14.8	65.1∗∗∗	19.6∗∗∗	6.5	3.9
Mother any DBM, child any DBM	8.4	12.9	15.4	53.8∗∗∗	19.8∗∗∗	5.4	4.0
Parent–child pairs, *n*	9,990	20,145	17,004				
Parent underweight, child overweight	1.0	0.6	0.9	−41.4∗∗∗	50.8∗∗∗	−4.1	10.2
Parent overweight, child undernourished	14.4	23.2	25.8	61.1∗∗∗	11.5∗∗∗	6.1	2.3
Parent any DBM, child any DBM	15.3	23.6	26.4	54.0∗∗∗	11.7∗∗∗	5.4	2.3

AACR, Average annual change rate DBM, double burden of malnutrition; NFHS, National Family Health Survey.

1 Percent change = $\left(\frac{Change}{Original}X\;100\right)$.

2 AACR = $\frac{Rate\;of\;change\;in\;the\;period}{Time\;Period}$ For example, in case of “Father underweight, child overweight”, the AACR was 14.6% $\left(\frac{Rate\;of\;change\;in\;the\;period}{Time\;Period}=\frac{73.1}{5}=14.6\right)$.

3 Child undernourished included child stunting and wasting. ∗∗∗*P* value < 0.000,∗∗*P* value < 0.10, ∗*P* value < 0.05 using *t*-statistics.

**FIGURE 2:**
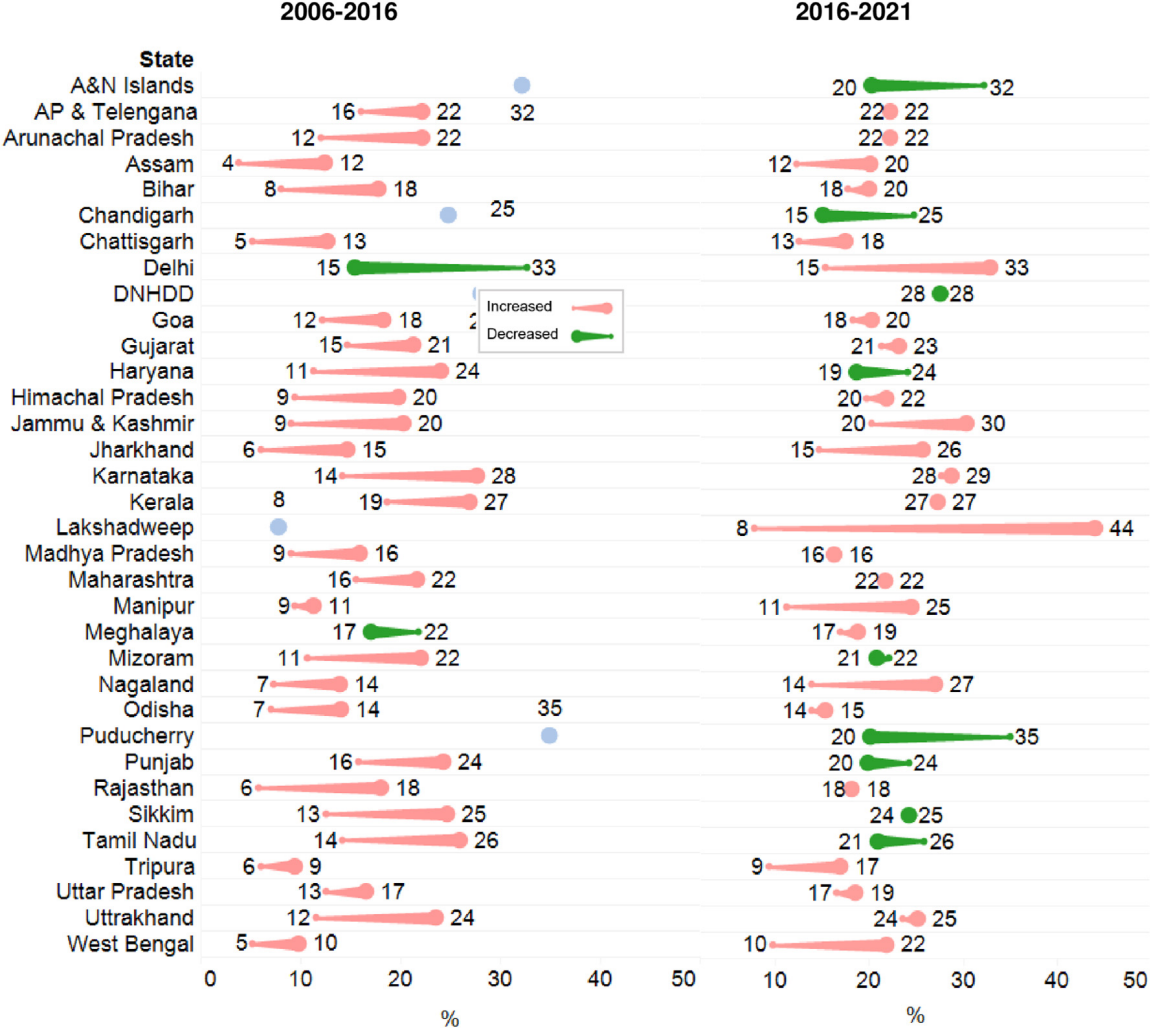
Subnational trends in the double burden of malnutrition among parent–child pair in India over time, National Family Health Survey (NFHS), 2006–2016 (A) and 2016–2021 (B). Comet tail (small dot) indicates earlier timepoint and comet head (large dot) indicates later timepoint. NFHS-3 (2006) does not provide estimates for UTs. A&N, Andaman and Nicobar; AP, Andhra Pradesh; DNHDD, Dadar and Nagar Haveli + Daman and Diu.

The prevalence of the DBM and trends among parents and children residing in the same household varied subnationally (Figure 2). The prevalence of the DBM ranged between 20% and 36% across Indian states and union territories (UTs) in 2021, with the highest prevalence observed in Chandigarh (43%), followed by Lakshadweep and Jammu Kashmir (35%–36%). Between 2006 and 2016, the prevalence of the DBM increased in all states except Delhi. The greatest increase was observed in the southern Indian state Karnataka (14 percentage points [pp]), followed by north-eastern states including Mizoram (11 pp) and Sikkim (12 pp). Between 2016 and 2021, in most states and UTs (*N* = 22/34), the prevalence of the DBM increased. The greatest increase was observed in the UT Lakshadweep (24 pp), followed by Nagaland (15 pp) and West Bengal (14 pp). In contrast, 12 out of 34 states and UT observed a reduction in DBM with the greatest decrease in UTs Puducherry (15 pp), Chandigarh, and Andaman and Nicobar Islands (12 pp). In father–child and mother–child dyads, the prevalence of the DBM ranged between 15% and 44% and 9% and 30%, respectively, in 2021 (Supplemental Figure 2).

### DBM pattern across residence

**FIGURE 3:**
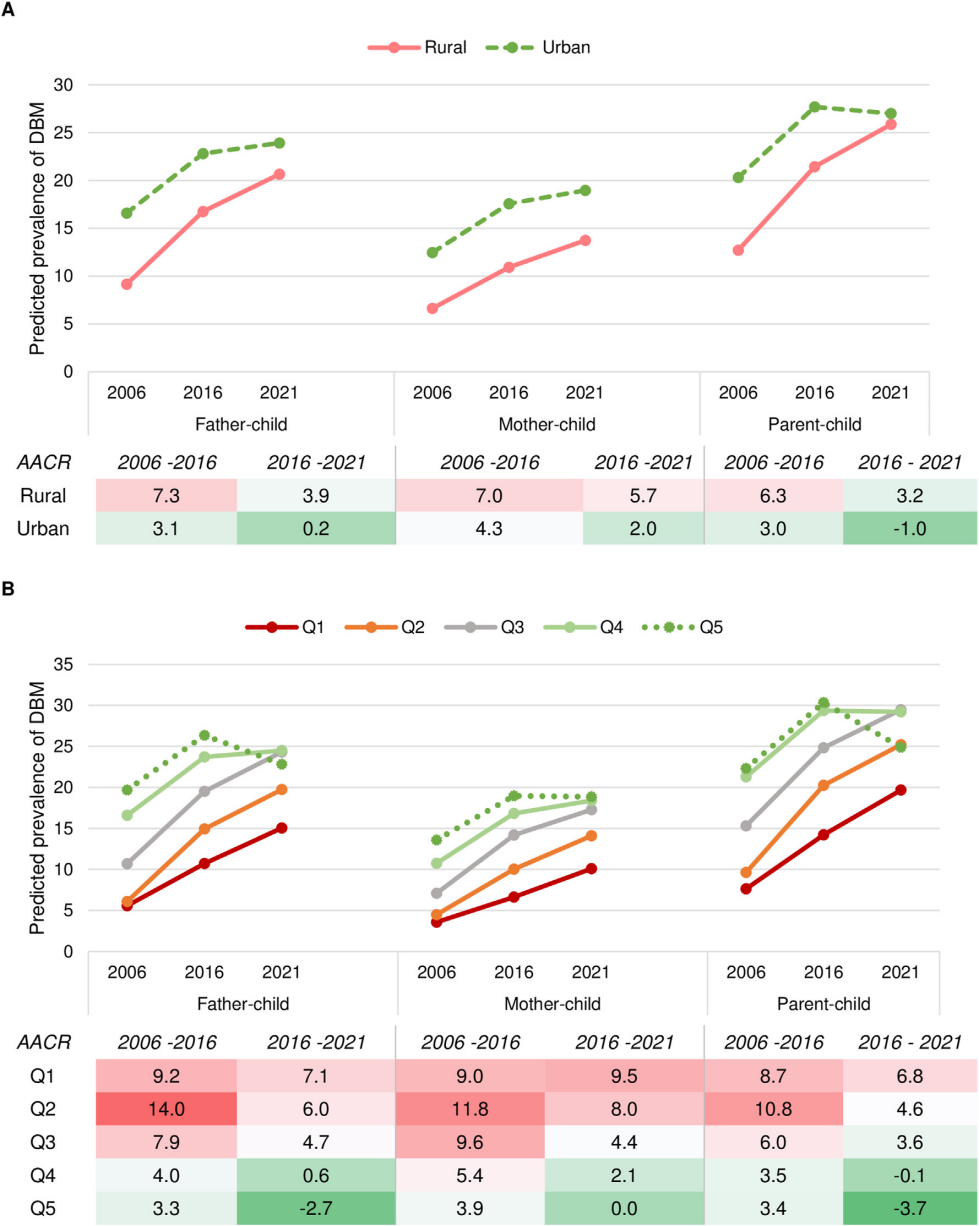
Predicted prevalence and average annual change rate of double burden of malnutrition in India by place of residence (A) and wealth quintile (B), National Family Health Survey (NFHS), 2006–2021.

The prevalence of the DBM in the parent–child dyad increased in rural areas from 13% to 26% between 2006 and 2021, with a higher AACR observed in 2006–2016 (6%) than in 2016–2021 (3%; **Figure 3**). In urban areas, the prevalence of the DBM increased from 20% to 28% between 2006 and 2016, but slightly declined to 27% in 2021. Although the prevalence of the DBM was higher in urban areas compared to rural areas across the 3 time periods, the rate of increase was higher in rural compared to urban areas (AACR 6% vs. 3% between 2006 and 2016, and 3% vs. −1% between 2016 and 2021). Similar patterns were observed in father–child and mother–child dyads where the prevalence of the DBM increased over time across residential areas, but the rate of change was higher in rural than in urban areas.

### DBM pattern across wealth quintiles

Between 2006 and 2016, the prevalence of the DBM increased across all quintiles, but the rate of change was higher among those belonging to the poorer quintile compared to the richer quintile (Figure 3). For example, between 2006 and 2016, AACR was 9% in quintile 1 but 3%–4% in quintile 5 for parents–child dyads. Between 2006 and 2021, the prevalence of the DBM continued to increase among quintile 1–3 households (AACR 4%–7%) but reduced in quintile 5 (AACR −4%) and was stagnant in quintile 4 households. By 2021, the prevalence of the DBM was nearly 30% in quintiles 3 and 4, 25% in quintiles 2 and 5, and 20% in quintile 1. Similar patterns were observed for other dyads as well.

## Discussion

Despite the accelerating rates of the double burden of malnutrition globally, empirical evidence on the intrahousehold burden remains limited in India [26]. We found that the intrahousehold DBM has worsened over time and affected 1 in 4 Indian households in 2021. Between 2016 and 2021, the rate of increase slowed down. The prevalence and trends in the DBM differed subnationally. The DBM increased in most states and UTs except Delhi between 2006 and 2016; DBM decreased in only 12 of 34 states and UTs during this period. Finally, although DBM prevalence was higher in urban and wealthier houses, it increased faster in rural and poorer households.

Gaps in the DBM between different wealth groups, areas of residence, and states have narrowed over time. Malnutrition has historically been related to poverty, but recent data reveal that the prevalence of overweight and obesity is growing among both rich and poor households [29]. We found that the DBM is higher in affluent urban families than in poorer rural homes. This might be attributable to an unequal distribution of living environment, job structure, food security, and nutritional and sedentary behaviors, all of which influence an individual’s anthropometric status [30]. Being overweight is more common among affluent and urban populations [31], and increasing overweight is the main driver of increasing DBM [27]. Current trends showed that overweight would rise much faster in rural regions than in urban areas [10,32]. In addition, findings from 103 LMICs (including India) showed that the burden of being overweight has grown over time among the poor income categories, which was formerly more prevalent among the highest wealth group [29]. Despite significant economic growth and food production improvements in India, food insecurity remains a pressing issue, affecting millions of households [33]. Although food insecurity is a well-known cause of undernutrition, it is also linked to overweight/obesity through several mechanisms [12]: *1*) affordability of energy-dense, processed foods; *2*) quantity and diversity of food consumed; *3*) spatial-temporal access to nutritious food (distance from food sources) and physical ability to travel that distance; *4*) interpersonal food choice and distribution (high level of social acceptance of energy-dense food and disparity in intrahousehold food distribution); and *5*) nondietary behaviors (physical activity and leisure time).

We found differences in DBM trends at the subnational level, which may reflect varying nutrition-related program coverage and quality. Large-scale nutrition schemes such as the Integrated Child Development Scheme (ICDS) [34,35] and Mid-Day Meal [36,37], which target relatively poor households, may partially explain the narrowing gap in DBM. The undernutrition gap between rural and urban areas has also been decreasing. Indirect nutrition improvement through improved access to healthcare, more knowledge of preventative healthcare, and government policies and measures to lower the illness burden may also contribute to the narrowing DBM gap between rich and poor households [37]. Examples of such programs are the Swachh Bharat Abhiyan [38], which aims to promote cleanliness and lower the prevalence of infectious illnesses, and the Ayushman Bharat plan [39], which strives to offer health insurance to disadvantaged groups.

Our study provides evidence of changes in DBM not only among mother–child pairs but also father–child and parent–child pairs. The most rapid DBM increase was observed in father–child pairs with an 83% increase between 2016 and 2021, mainly driven by increasing overweight among fathers. Therefore, a family-centered approach that includes fathers should be implemented to strengthen existing programs addressing DBM in India. Traditionally, discussions on nutrition in Indian households have focused on the roles of mothers and their caregiving practices [18]. However, it is imperative to recognize that fathers play a crucial role in shaping the family’s nutrition environment because they have influence over decision-making processes, resource allocation, and behaviors pertaining to food and nutrition [15]. Engaging fathers in decision-making can positively influence the selection and procurement of healthier food options, particularly for poor households [40]. Fathers can increase family access to nutritious food, which in turn influence household dietary intake and nutritional status [[17], [18], [19]]. Fathers can also support healthy lifestyle choices by encouraging physical activity and limiting the consumption of unhealthy foods, which lead lower rates of childhood obesity [41].

The study uses 3 rounds of the NFHS’s nationally representative dataset to examine trends in the DBM by geography, residence type, and wealth. Few studies have examined the DBM within families, and thus, the study fills an important research gap. However, our study is not without limitations. The estimates are based on cross-sectional surveys of different households at each timepoint, and hence, we do not attempt to causally identify factors driving the increase in DBM.

In order to address the DBM in India, continued efforts are required to reduce undernutrition, particularly in rural and poorer households, while simultaneously addressing rising rates of overweight and obesity in richer households, particularly in urban areas [10]. Interventions should focus on boosting diet diversity, making good food choices, and increasing physical activity levels [42]. Rapid urbanization in India necessitates policies that prioritize improving urban food systems, diets, and lifestyles. Projections suggest that adult obesity in India will more than double between 2010 and 2040 [29]. Multisectoral strategies that take into account the socioeconomic, cultural, and environmental aspects, including family-centered approach and father involvement, are needed to address multiple forms of malnutrition simultaneously. Examples of such strategies including front-of-package labeling to reduce unhealthy food consumption [43], increasing support to mothers to promote appropriate breastfeeding and complementary feeding, strengthening growth monitoring programs, establishing regular check-ups for all household members for early detection of overnutrition, educating families about meal planning and healthy diets, and offering farmers incentives to produce more nutritious foods [44]. Additional efforts are needed to address food insecurity at the household level, to ensure access to nourishing and diverse foods, and to promote healthy eating habits.

In conclusion, it is unacceptable for 1 in 4 Indian households with children aged <5 y to have both an overweight parent and an underweight child. In order to achieve Sustainable Development Goals 2 related to nutrition, health, and well-being, the DBM must be addressed. Coordinated efforts from all stakeholders are required to reduce the intrahousehold DBM in India.

## Author contributions

The authors’ responsibilities were as follows – LKD: conceptualized the study and wrote the manuscript, reviewed, and edited the manuscript; PP: curated the data, performed data analysis, wrote the manuscript, and reviewed and edited the manuscript; AP: performed data visualization, wrote the manuscript, and reviewed and edited the manuscript; AC; reviewed and edited the manuscript; SS: wrote the manuscript, and reviewed and edited the manuscript; SKS: reviewed and edited the manuscript; SP: reviewed and edited the manuscript; PHN: acquired funding, conceptualized the study, wrote the manuscript, and reviewed and edited the manuscript; and all authors: read and approved the final manuscript. The views expressed in this paper are solely those of the authors and do not necessarily represent those of the organization to which the authors belong.

## Conflict of interest

The authors report no conflicts of interest.

## Funding

This study was supported by Bill & Melinda Gates Foundation, through POSHAN, led by the International Food Policy Research Institute. The funding has no role in the study design, collection, analysis, interpretation of data, writing of the report, or decision to submit for publication.

## Data Availability

All data used in the study is archived in the public repository of Demographic and Health Survey (DHS). The data can be accessed using https://dhsprogram.com/data/dataset_admin/index.cfm, which requires registration.

## Ethics approval

The study was exempted by the Institutional Review Board held at the International Institute for Population Sciences (IIPS), Mumbai, India, because the data used in the study were from secondary source in the public domain.

## Declaration of interests

The authors declare that they have no known competing financial interests or personal relationships that could have appeared to influence the work reported in this paper.
