# Supplementary material for: Concurrent Undernutrition and Overnutrition within Indian Families between 2006 and 2021
Source: Curr Dev Nutr. 2023 Aug 19;7(9):101987. doi: 10.1016/j.cdnut.2023.101987 (PMC10502368; doi:10.1016/j.cdnut.2023.101987)
Supplement: Multimedia component 1 [file mmc1.docx]

**Supplemental Figure 1:** Prevalence of selected indictors of malnutrition among (A) children below five years (B) mothers (15-49 years) and (C) fathers (15-54 years) in India, National Family Health Survey (NFHS), 2006-21.

**
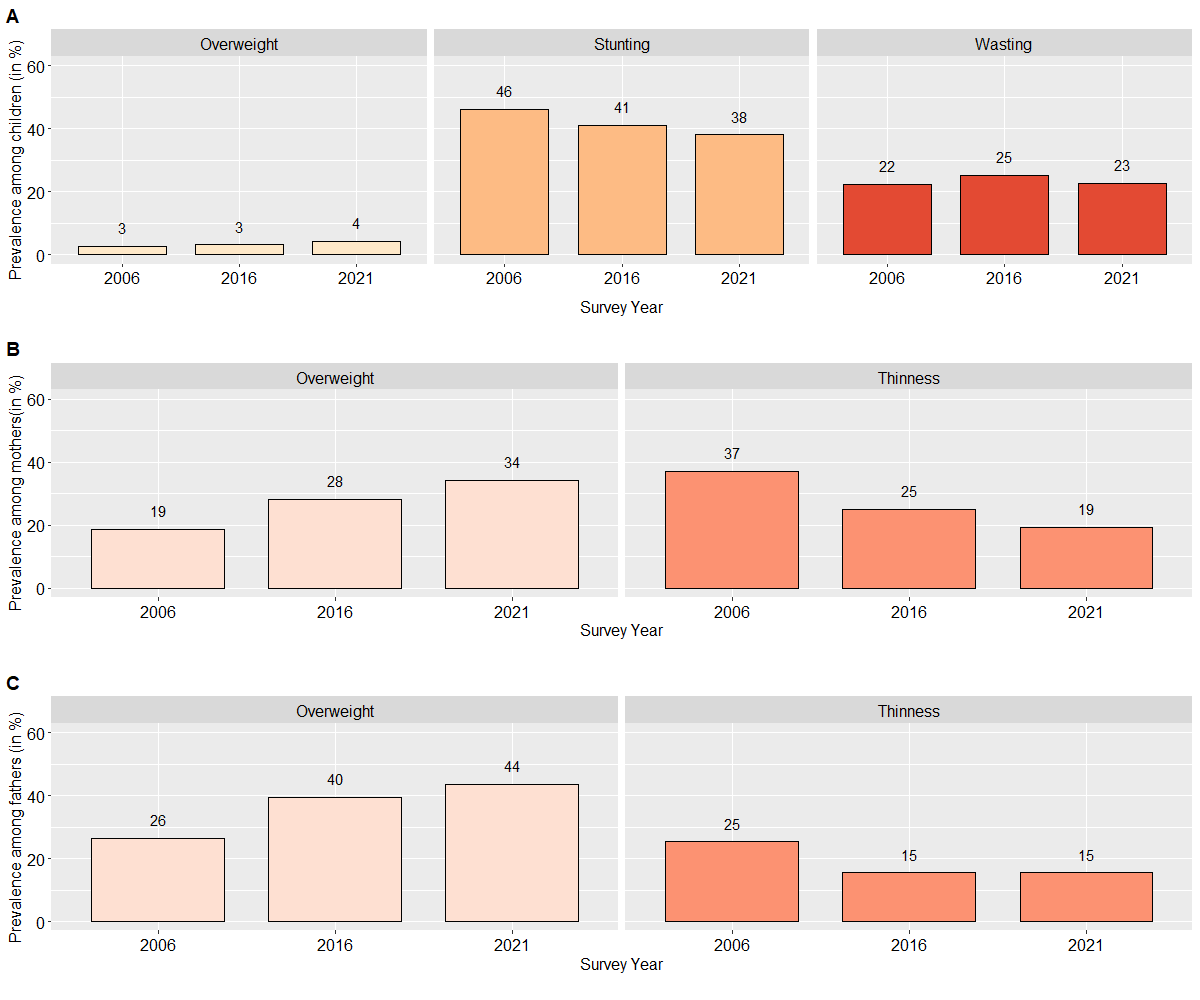
**

**For children,** stunting was defined as height-for-age Z-scores (HAZ) below minus two standard deviations (-2 SD) from the median of the reference population. Wasting was defined as weight-for-height Z-scores (WHZ) below minus two standard deviations (-2 SD) from the median of the reference population, respectively. Overweight/obesity was defined as weight-for-height Z-scores (WHZ) above two standard deviations (+2 SD).

**For adults**, height and weight were used to calculate Body Mass Index (BMI), then categorized to underweight (BMI < 18.5 kg/m^2^ ) or overweight/obesity (BMI > 23 kg/m^2^) based on recommendation for Asian population.

Supplemental Figure 2: Sub-national trends in the double burden of malnutrition among (a) father-child and (b) mother-child pair in India over time, National Family Health Survey (NFHS), 2006-21

A.

| **2006-2016**   **2016-2021** |
| --- |
| 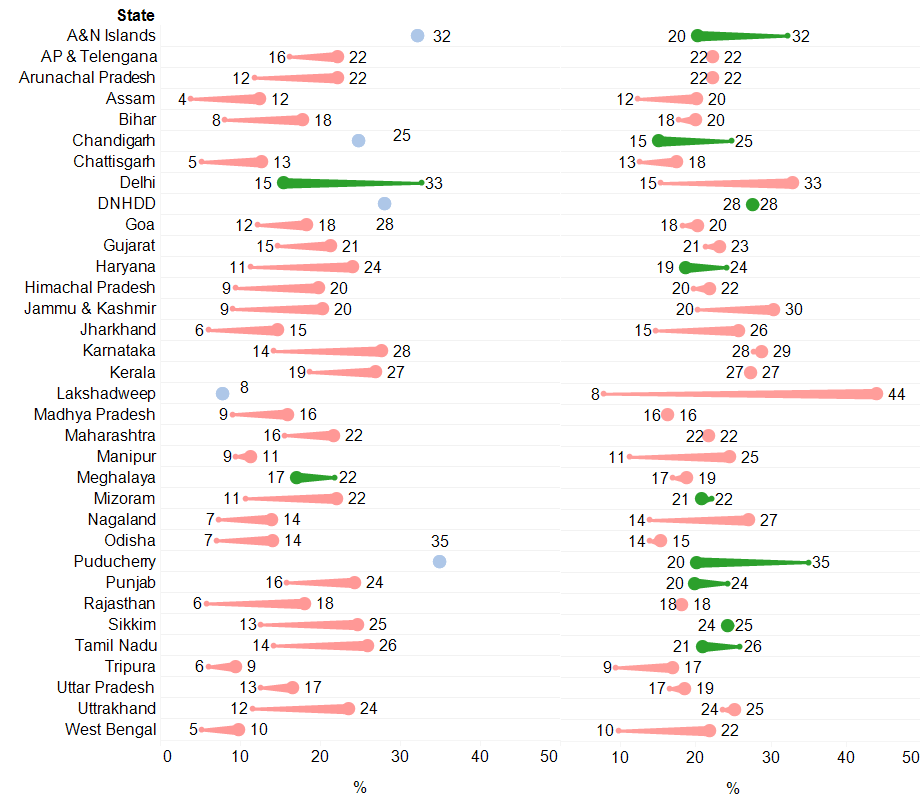 |
| **B.**  **2006-2016**   **2016-2021** |
| 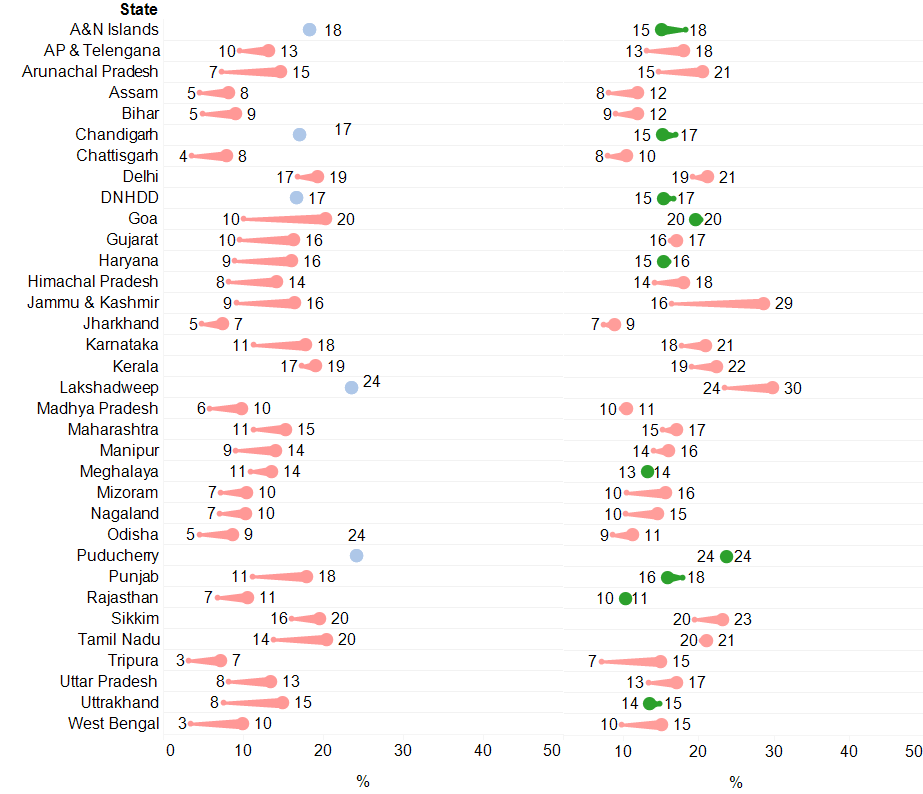 |
|  |
